# Natural killer cell activity is a risk factor for the recurrence risk after curative treatment of hepatocellular carcinoma

**DOI:** 10.1186/s12876-021-01833-2

**Published:** 2021-06-12

**Authors:** Han Ah Lee, Hyun Gil Goh, Young-Sun Lee, Young Kul Jung, Ji Hoon Kim, Hyung Joon Yim, Min-Goo Lee, Hyunggin An, Yoon Tae Jeen, Jong Eun Yeon, Kwan Soo Byun, Yeon Seok Seo

**Affiliations:** 1 Department of Internal Medicine, Korea University College of Medicine, 73, Goryeodae-ro, Seongbuk-Gu, Seoul, Korea; 2 Department of Internal Medicine, Sanggye Paik Hospital, Inje University College of Medicine, Seoul, Korea; 3 Department of Physiology, Korea University College of Medicine, Seoul, Korea; 4 Department of Biostatistics, Korea University College of Medicine, Seoul, Korea

**Keywords:** Natural killer cell, Interferon-γ, Hepatocellular carcinoma, Stage, Recurrence

## Abstract

**Background:**

Natural killer (NK) cells have been known to contribute to surveillance and control of hepatocellular carcinoma (HCC). However, the association of NK cell activity with stage and recurrence risk of HCC have not been fully evaluated.

**Methods:**

Untreated patients with newly diagnosed HCC were prospectively enrolled. Peripheral blood mononuclear cells were isolated at the time of diagnosis. Patients who had undergone surgery or radiofrequency ablation were classified as the curative treatment group, and their blood samples were collected again at 1 month after treatment.

**Results:**

A total of 80 patients with HCC were enrolled. The mean age was 62.5 years. At baseline, interferon (IFN)-γ producing NK cell proportion was significantly lower in patients with Barcelona clinic liver cancer (BCLC) stage B, C, or D than in those with BCLC stage 0 (42.9% vs. 56.8%, *P* = 0.045). Among all patients, 56 patients had undergone curative treatment, and 42 patients re-visited at 1 month after curative treatment. There was no significant change in total NK cell and IFN-γ producing NK cell proportion from baseline to 1 month after treatment (all *P* > 0.05). During a median follow-up of 12.4 months, HCC recurred in 14 patients (33.3%). When patients were classified according to the IFN-γ producing NK cell proportion (group 1, ≥ 45%; and group 2, < 45%), HCC recurrence rate did not differ according to the IFN-γ producing NK cell proportion at baseline (log-rank test, *P* = 0.835). However, patients with < 45% IFN-γ producing NK cell proportion at 1 month after treatment had a significantly higher HCC recurrence rate than patients with that of ≥ 45% (log-rank test, *P* < 0.001). Multivariate analysis revealed that BCLC stage B (hazard ratio [HR] = 3.412, *P* = 0.045) and < 45% IFN-γ producing NK cell proportion at 1 month after treatment (HR = 6.934, *P* = 0.001) independently predicted an increased risk of HCC recurrence.

**Conclusions:**

Decreased NK cell activity is significantly associated with the advanced stage of HCC, and the increased recurrence risk of HCC after curative treatment.

**Supplementary Information:**

The online version contains supplementary material available at 10.1186/s12876-021-01833-2.

## Background

Hepatocellular carcinoma (HCC) is currently the second leading cause of cancer-related mortalities worldwide [1], and have poor prognosis with high recurrence rate even in patients with early stage HCC treated with curative modality [2–5]. Since recurrence of HCC is the main cause of mortality [6, 7], identifying significant predictors for recurrence and stratifying risk of recurrence are important in managing patients with HCC.

Because most tumors arise in hepatic inflammation and consequent fibrosis, HCC appears as an inflammation-associated malignancy, characterized by infiltration of diverse immune cells [8–11]. It has been indicated that immune cells in the tumor microenvironment play a critical role in defense against HCC progression [12, 13]. The first host defense against tumor is innate immunity, and the liver contains a substantial number of various innate lymphocytes including natural killer (NK) cells [14, 15]. NK cells account for a large proportion of innate lymphocytes, and are significantly involved in innate and adaptive immunological defense against cancer development [16].

Naturally, NK cells contribute to surveillance and control of HCC, capable of killing cancer cells. However, NK cells lose their function in the tumor microenvironment via various mechanisms: attenuation of absolute numbers, defective cytokine secretion, abnormal expression of NK cell receptors, and inhibition of NK cells by other immunoregulatory cells [17]. Dysfunction of NK cells has been suggested as an important mechanism for evasion of tumor cells [18]. Accordingly, several studies have suggested the relationship between multiplicity and function of NK cells and prognosis of patients with HCC [19–22]. Furthermore, previous studies in patients with chronic hepatitis C and hematologic malignancy showed that the function of NK cells recovered after antiviral therapy or chemotherapy, and the recovery of NK cell activity was correlated with improved prognosis [23–26]. However, because of only a limited number of studies have assessed the number and function of NK cells after treatment, the changes and significance of NK cell activity after curative treatment have not been fully evaluated.

Thus, we performed this study with untreated patients with newly diagnosed HCC based on the following hypotheses: (1) NK cell activity at diagnosis would be significantly associated with the stage of HCC. (2) NK cell activity may be altered after curative treatment in some patients with HCC. 3) NK cell activity after curative treatment may be an independent predictor for prognosis in HCC patients. A better understanding of these issues would contribute to the development of successful immunotherapies and selection of patients with unrestored immune function, who are indicated to have immunologic treatment after curative treatment of HCC.

In summary, we recruited patients with newly diagnosed HCC and assessed the proportion and activity of NK cells before and after curative treatment. In addition, we investigated the association of NK cell activity with HCC stage and the recurrence after curative treatment.

## Methods

### Patients and specimens

Untreated patients with newly diagnosed HCC were prospectively enrolled between 2016 and 2018 from Korea University Medical Center. The exclusion criteria were (1) age < 18 years; (2) history of HCC or organ transplant; (3) history of HCC treatment; (4) use of immunosuppressive agents; and (5) any other significant medical illness. Finally, a total of 90 patients were eligible and followed until September 2019. Patients were divided into two groups according to the treatment modality: (1) patients treated with surgical resection or radiofrequency ablation (RFA) (curative treatment group) and (2) patients treated with therapies other than resection or RFA, including best supportive care (conservative treatment group) (Additional file 1: Fig. 1).

Peripheral blood samples were obtained from all enrolled patients at the time of diagnosis. Among the 90 enrolled patients, 10 patients were excluded from the final analysis because of poor quality of peripheral mononuclear cells (PBMCs). Patients in the curative treatment group re-visited our clinic at 1 month after curative treatment, and their blood samples were collected again. Heparin tubes (BD Biosciences, Franklin Lakes, NJ, USA) were used to collect blood samples.

### Isolation of mononuclear cells from peripheral blood and tissues

PBMCs were isolated from collected blood by Ficoll density gradient centrifugation (Biochrom, Berlin, Germany) as previously described [27, 28]. The mononuclear cells were washed and resuspended in medium supplemented with 1% heat‐inactivated fetal calf serum (BD Biosciences) for fluorescent‐activated cell sorter (FACS) analysis.

### Flow cytometry analysis

**Fig. 1:**
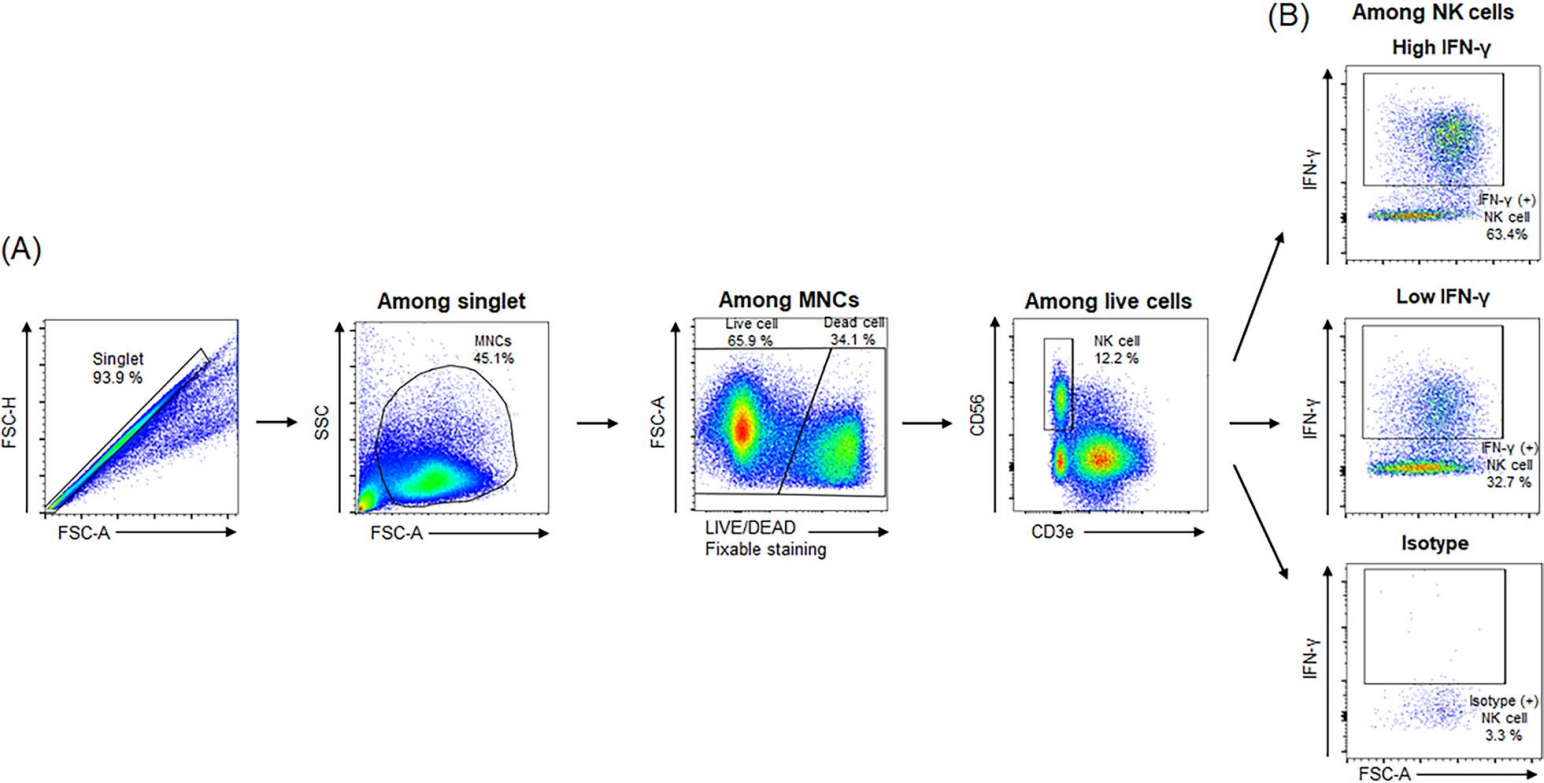
The gating process of flow cytometric analysis (**A**) and IFN-γ production of NK cell with high IFN-γ (+) NK cell, low IFN-γ (+) NK cell, and isotype control (**B**). FSC-A, forward scatter area; FSC-H, forward scatter height; SSC, side scatter; MNCs, mononuclear cells; CD, cluster of differentiation; NK, natural killer, IFN-γ, interferon gamma

Leukocytes were stained with surface markers, fixed, permeabilized with IntraPre Reagent (Beckman Coulter, Fullerton, CA, USA), and further stained with the following antibodies against intracellular markers: anti-CD3 monoclonal antibody (mAb), anti-CD56 mAb, and anti-IFN-γ mAb (BD Biosciences). The FlowJo software program (version 10, FLOWJO, BD Biosciences) was used for data analysis. For the measurement of intracellular cytokine production, cells were stimulated at 37 °C for 5 h with Leukocyte Activation Cocktail (BD Biosciences) before staining, as previously described [29]. When we checked cell viability after thawing of frozen cells, the viability were good with more than 75%. Cell viabilities were approximately checked 65% using dead cell marker. (Additional file 2: Fig. 2). We analyzed IFN-γ positive NK cell population and the gating process was summarized at Fig. 1A. Finally, T-cell producing IFN-γ was analyzed (Additional file 3: Fig. 3).

### Clinical evaluation and follow-up

The index date was defined as the date of HCC diagnosis. Diagnosis of HCC was based on non-invasive criteria and/or pathology in cirrhotic patients, and pathology in non-cirrhotic patients. Non-invasive criteria were based on the identification of typical hallmarks of HCC, obtained by multiphasic computed tomography (CT) or multiphase magnetic resonance imaging (MRI) (nodule > 1 cm with arterial hypervascularity and portal/delayed-phase washout) [30–32]. Liver cirrhosis was diagnosed when typical ultrasonographic findings were found, together with a low platelet count (< 100,000/μL), varices, or overt complication of cirrhosis [33].

### Treatment and recurrence surveillance after curative treatment

The treatment type was determined by the clinician based on the clinical practice guideline for HCC in Korea [34]. In patients in the curative treatment group, treatment response was evaluated at 1 month after treatment, and complete response was defined as the disappearance of any intratumoral arterial enhancement in all target lesions, as evaluated by multiphasic CT scan or MRI at 1 month after treatment [35]. Patients that underwent curative treatment were surveilled periodically after treatment. Surveillance for recurrence was performed using imaging techniques, such as multiphasic CT scan or MRI and blood tests including tumor markers. Owing to the lack of evidence-based guidelines for recurrence surveillance, screening was conducted every 1–6 months as per the clinician’s decision.

### Outcomes

The primary outcome was the recurrence of HCC. The secondary outcome was the stage of HCC at diagnosis. Intrahepatic HCC recurrence was defined by the same criteria applied for initial HCC diagnosis. Extrahepatic recurrence was evaluated by CT or bone scan performed at the discretion of the clinician. Barcelona clinic liver cancer (BCLC) staging system was used for HCC staging [36].

### Statistical analysis

Results are expressed as means ± standard deviations or numbers with percentages. The statistical significance of differences between continuous and categorical variables was examined by Student’s *t*-test (or the Mann–Whitney test, when appropriate) and the chi-squared test (or Fisher’s exact test, when appropriate), respectively. The cumulative incidences of HCC recurrence were calculated using the Kaplan–Meier method with time-to-progression. These incidences were compared among subgroups of the study population defined based on the NK cell profile using the log-rank test. Multivariate analyses were performed to identify independent predictors of HCC recurrence using the Cox proportional hazards model. SPSS statistical software (version 13.0) was used for all statistical analyses. All data were analyzed using two-tailed tests unless otherwise specified, and *P* < 0.05 was considered statistically significant.

## Results

### Baseline characteristics of the study population

A total of 80 patients were finally selected for the statistical analysis (Additional file 1: Fig. 1). The baseline characteristics of the study population are presented in Table 1. The mean age of the patients was 62.5 (56.0–70.0) years, and 60 patients (75.0%) were men. Chronic hepatitis B virus (HBV) infection was the most frequent underlying liver disease (48 patients, 60.0%). Liver cirrhosis was combined in 64 patients (80.0%). Because of the small numbers of patients in BCLC stage C and D, patients were classified into 3 groups: BCLC 0, patients with BCLC stage 0 (n = 15, 18.8%); BCLC A, patients with BCLC stage A (n = 38, 47.5%); and BCLC BCD, patients with BCLC stage B, C, or D (27 patients, 33.8%).

**Table 1:** Baseline characteristics of the study population according to the BCLC stage

Variables	All patients (n = 80)	BCLC 0 (n = 15, 18.8%)	BCLC A (n = 38, 47.5%)	BCLC BCD (n = 27, 33.7%)
Age, years	62.5 (56.0–70.0)	63.3 (58.5–69.0)	64.2 (57.5–71.0)	59.6 (53.0–66.5)
Male gender	60 (75.0)	10 (66.7)	30 (78.9)	20 (74.1)
Etiology				
Hepatitis B virus	48 (60.0)	9 (60.0)	20 (52.6)	19 (70.4)
Hepatitis C virus	7 (8.8)	2 (13.3)	4 (10.5)	1 (3.7)
Alcohol	13 (16.2)	1 (6.7)	9 (23.7)	3 (11.1)
Others	12 (15.0)	3 (20.0)	5 (13.2)	4 (14.8)
Liver cirrhosis	64 (80.0)	12 (80.0)	28 (73.7)	24 (88.9)
Child–Pugh class				
A	62 (77.5)	15 (100)	31 (81.6)	16 (59.3)
B or C	18 (22.5)	0 (0)	7 (18.4)	11 (40.7)
Total bilirubin, mg/dL	1.44 (0.70–1.20)	0.67 (0.35–0.95)	1.14 (0.70–1.15)	2.24 (0.80–2.05)
Albumin, g/dL	3.9 (3.4–4.3)	4.0 (3.5–4.5)	3.9 (3.5–4.5)	3.7 (3.4–4.1)
Aspartate aminotransferase, IU/L	70.7 (33.0–80.0)	48.9 (23.5–56.0)	48.1 (31.0–59.5)	111.8 (54.5–126.5)
Alanine aminotransferase, IU/L	44.6 (19.0–52.0)	31.5 (14.0–42.0)	37.7 (18.5–44.5)	60.4 (28.5–54.5)
Platelet count, 10^9^/L	144.8 (97.0–181.0)	126.9 (76.0–155.0)	144.8 (87.5–195.0)	154.1 (116.5–185.0_
Prothrombin time, INR	1.12 (1.03–1.17)	1.08 (0.99–1.14)	1.11 (1.01–1.20)	1.14 (1.04–1.22)
Alpha-fetoprotein, ng/mL	7620.9 (4.4–439.8)	91.0 (2.4–61.2)	3712.8 (3.6–74.6)	16,695.1 (92.5–69,082.0)
PIVKA-II, mAU/mL	6918.1 (18.0–649.0)	32.9 (58.5–69.0)	506.9 (57.5–71.0)	18,961.3 (53.0–66.5)
Type of treatment				
Resection	31 (38.8)	7 (46.7)	18 (47.4)	6 (22.2)
Radiofrequency ablation	25 (31.2)	6 (40.0)	18 (47.4)	1 (3.7)
Transarterial chemoembolization	8 (10.0)	2 (13.3)	2 (5.3)	4 (14.8)
Sorafenib	5 (6.2)	0 (0)	0 (0)	5 (18.5)
Radiation therapy	1 (1.2)	0 (0)	0 (0)	1 (3.7)
Transarterial radioembolization	3 (3.8)	0 (0)	0 (0)	3 (11.1)
Conservative treatment	7 (8.8)	0 (0)	0 (0)	7 (25.9)

Variables are expressed as mean (interquartile range) or n (%). *BCLC* Barcelona clinic liver cancer, *BCLC 0* patients with BCLC stage 0, *BCLC A* patients with BCLC stage A, *BCLC BCD* patients with BCLC stage B, C, or D

### ***IFN-***γ*** producing NK cell proportion significantly decreased in advanced-stage HCC***

**Fig. 2:**
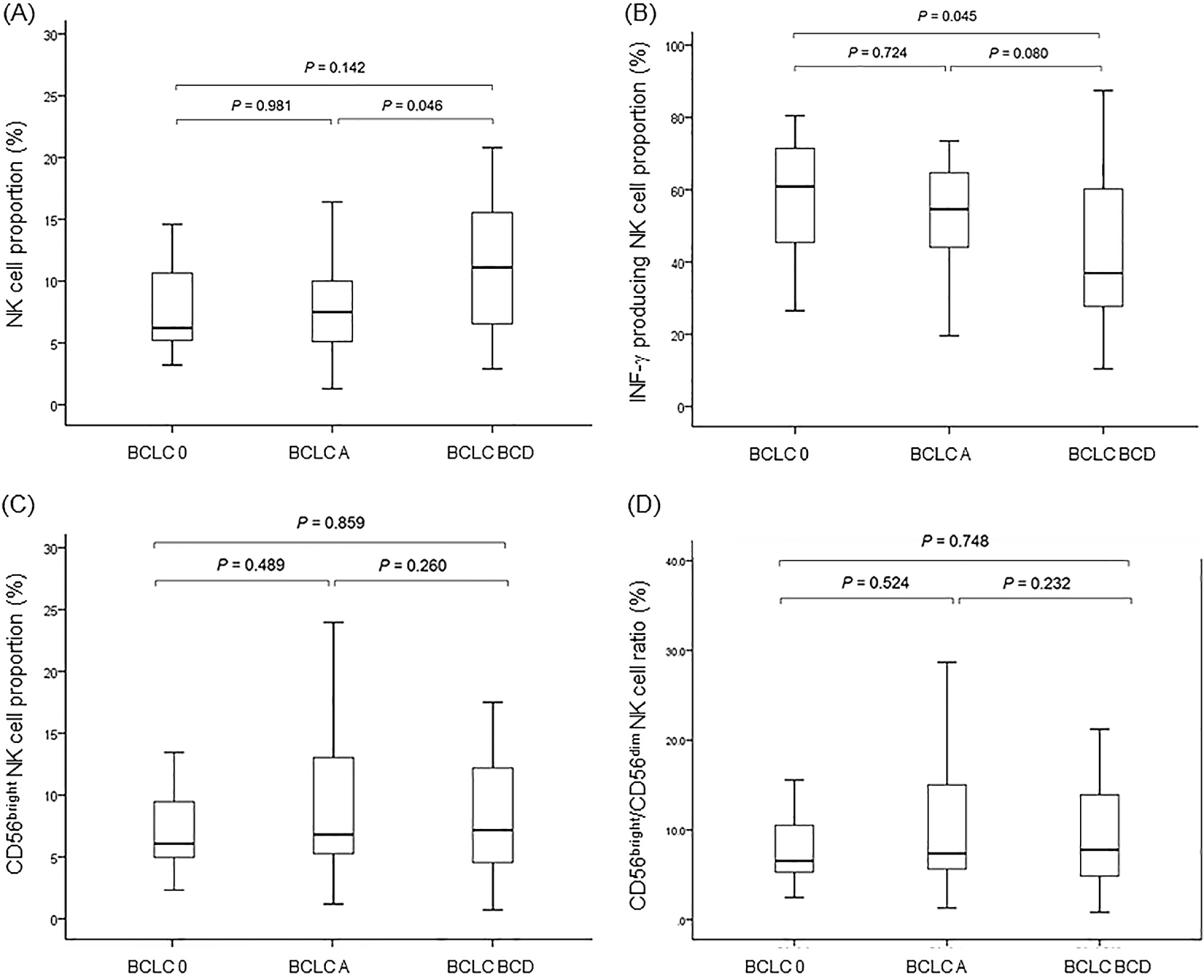
NK cell proportion (**A**), IFN-γ producing NK cell proportion (**B**), CD56^bright^ NK cell proportion (**C**), and ratio of CD56^bright^ NK cell and CD56^dim^ NK cell (**D**) according to the BCLC stage. NK, natural killer; IFN, interferon; BCLC, Barcelona clinic liver cancer; BCLC 0, patients with BCLC stage 0; BCLC A, patients with BCLC stage A; and BCLC BCD, patients with BCLC stage B, C, or D

As shown in Fig. 2A, the total NK cell proportion was significantly higher in patients with BCLC BCD than in those with BCLC A (12.4% vs. 8.6%, *P* = 0.046). IFN-γ producing NK cell proportion was significantly lower in patients with BCLC BCD than in those with BCLC 0 (42.9% vs. 56.8%, *P* = 0.045), and there was a trend of lower IFN-γ producing NK cell proportion in patients with BCLC BCD than in those with BCLC A (42.9% vs. 52.6%, *P* = 0.080) (Fig. 2B). There were no statistical differences in T-cell producing IFN-γ proportion according to BCLC stages (all P > 0.05). CD56^bright^ NK cell proportion and ratio of CD56^bright^ NK cell and CD56^dim^ NK cell were comparable among all groups (all *P* > 0.05) (Fig. 2C, D).

### Baseline characteristics of patients in the curative treatment group

Of the 56 patients in the curative treatment group, 42 patients re-visited at 1 month after curative treatment, and their blood samples were collected again. The baseline characteristics of the curative treatment group are presented in Table 2. There were 13 patients (31.0%) in BCLC stage 0, 21 patients (50.0%) in BCLC stage A, and 8 patients (19.0%) in BCLC stage B. Patients were treated with surgical resection (28 patients, 66.7%) or RFA (14 patients, 33.3%).

**Table 2:** Characteristics of the population according to the post 1 month IFN-γ producing NK cell proportion

Variables	All patients (n = 42)	Group 1, IFN-γ producing NK cell proportion ≥ 45% (n = 26, 61.9%)	Group 2, IFN-γ producing NK cell proportion < 45% (n = 16, 38.1%)	*P* value
Age, years	62.6 (65.5–68.3)	62.5 (56.3–68.5)	62.1 (55.8–68.8)	0.889
Male gender	29 (69.0)	15 (57.7)	13 (81.2)	0.116
Etiology				0.082
Hepatitis B virus	25 (59.5)	13 (50.0)	12 (75.0)	
Hepatitis C virus	4 (9.5)	4 (15.4)	0 (0.0)	
Alcohol	7 (16.7)	3 (11.5)	4 (25.0)	
Others	6 (14.3)	6 (23.1)	0 (0.0)	
Liver cirrhosis	32 (76.2)	19 (73.1)	13 (81.2)	0.546
Child–Pugh class				0.969
A	34 (81.0)	21 (80.8)	13 (81.2)	
B	8 (19.0)	5 (19.2)	3 (18.8)	
BCLC stage				0.897
0	13 (31.0)	7 (26.9)	6 (37.5)	
A	21 (50.0)	14 (53.8)	7 (43.8)	
B	8 (19.0)	5 (19.3)	3 (18.7)	
Total bilirubin, mg/dL	1.07 (0.60–1.10)	1.15 (0.60–1.10)	0.94 (0.40–1.65)	0.433
Albumin, g/dL	4.1 (3.7–4.5)	4.1 (3.6–4.5)	4.1 (3.6–4.6)	0.796
Aspartate aminotransferase, IU/L	50.0 (25.0–61.5)	53.9 (26.8–62.3)	40.6 (24.0–60.0)	0.206
Alanine aminotransferase, IU/L	37.1 (19.0–51.0)	39.5 (19.0–56.8)	28.6 (17.0–40.0)	0.098
Platelet count, 10^9^/L	147.5 (87.5–195.0)	145.2 (81.5–210.8)	155.9 (89.5–183.5)	0.598
Prothrombin time, INR	1.09 (1.00–1.16)	1.13 (1.05–1.16)	1.06 (0.99–1.17)	0.192
Alpha-fetoprotein, ng/mL	3293.4 (3.6–72.0)	40,702.0 (3.8–63.7)	1003.8 (3.4–481.1)	0.546
PIVKA-II, mAU/mL	992.5 (14.0–102.0)	621.2 (14.0–79.3)	1678.2 (16.5–264.0)	0.432
Type of treatment				0.822
Resection	28 (66.7)	17 (65.4)	11 (68.8)	
Radiofrequency ablation	14 (33.3)	9 (34.6)	5 (31.2)	

Variables are expressed as mean (interquartile range) or n (%). *BCLC* Barcelona clinic liver cancer, *IFN* interferon

### NK cell profiles were not significantly changed after curative treatment

There were no significant changes in total NK cell proportion (9.6% vs. 11.2%, *P* = 0.299) and IFN-γ producing NK cell proportion (50.5% vs. 45.1%, *P* = 0.108) from baseline to 1 month after curative treatment (Additional file 4: Fig. 4). In addition, the proportions of CD56^bright^ NK cell and CD56^dim^ NK cells were also not significantly changed from baseline to 1 month after curative treatment (all *P* > 0.05).

### ***IFN-***γ*** producing NK cell proportion at 1 month after curative treatment significantly predicted HCC recurrence***

Among 42 patients who re-visited at 1 month after curative treatment, HCC recurred in 14 patients (33.3%) during the median follow-up of 12.4 months. HCC recurrence rates at 6, 12, 18, and 24 months were 12.7%, 28.1%, 41.2%, and 41.2%, respectively. To investigate the clinical significance of NK cell activity in HCC recurrence, patients were classified into 2 groups according to the IFN-γ producing NK cell proportion as follows: group 1, patients with IFN-γ producing NK cell proportion ≥ 45%; and group 2, patients with IFN-γ producing NK cell proportion < 45% (Fig. 1B). The number of patients with ≥ 45% IFN-γ producing NK cell proportion was 28 (66.6%) at baseline, and 26 (61.9%) at 1 month after treatment.

**Fig. 3:**
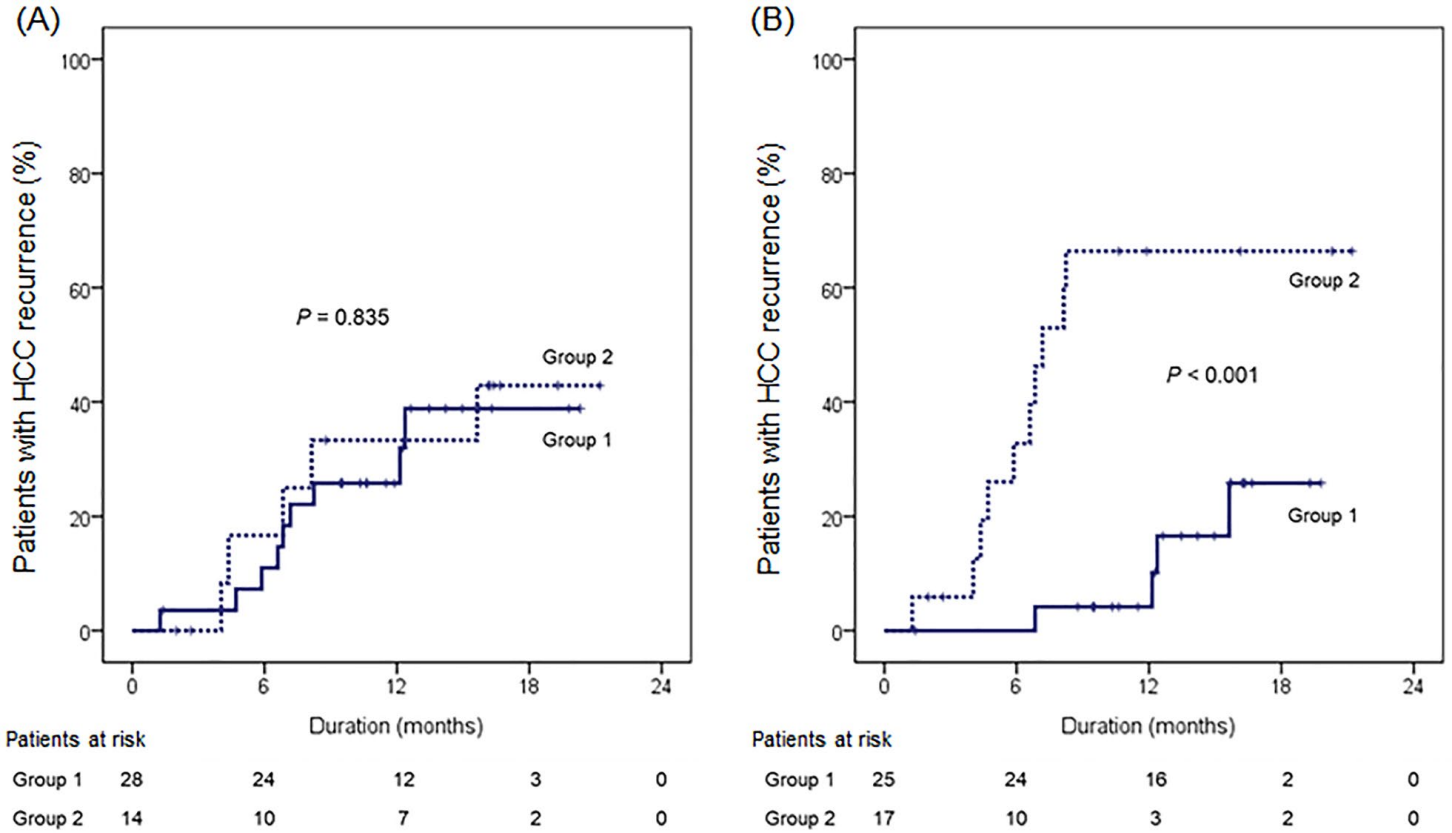
HCC recurrence rates among groups according to the IFN-γ producing NK cell proportion at baseline (**A**) and 1 month after treatment (**B**). Group 1, patients with IFN-γ producing NK cell proportion ≥ 45%; Group 2, patients with IFN-γ producing NK cell proportion < 45%. HCC, hepatocellular carcinoma; IFN, interferon; NK, natural killer

There was no significant difference in HCC recurrence rate between patients with < 45% IFN-γ producing NK cell proportion at baseline and patients with that of ≥ 45% (*P* = 0.835) (Fig. 3A). However, patients with < 45% IFN-γ producing NK cell proportion at 1 month after treatment had a significantly higher HCC recurrence rate than patients with that of ≥ 45% (*P* < 0.001) (Fig. 3B). There were no other significant difference in baseline characteristics between patients in group 1 and 2, according to the IFN-γ producing NK cell proportion at 1 month after treatment (all *P* > 0.05) (Table 2). Based on the striking positive association between IFN-γ producing NK cell proportion at 1 month after curative treatment and HCC recurrence, further multivariate analysis revealed that BCLC stage B (hazard ratio [HR] = 3.412, 95% confidence interval [CI] 1.026–11.355; *P* = 0.045) and < 45% IFN-γ producing NK cell proportion at 1 month after treatment (HR = 6.934, 95% CI 2.100–22.897; *P* = 0.001) independently predicted an increased risk of HCC recurrence (Table 3). T-cell producing IFN-γ proportion at baseline (HR 0.976, *P* = 0.448), and 1 month after curative treatment (HR 0.949, *P* = 0.325) were not associated with the risk of HCC recurrence.

**Table 3:** Predictors for HCC recurrence

Variable	Rating	Univariate	Multivariate		
		*P* value	HR	95% CI	*P* value
Age	Years	0.549			
Sex	0 = female; 1 = male	0.497			
Etiology	1 = viral; 2 = other	0.907			
Liver cirrhosis	0 = no; 1 = yes	0.0578			
Type of treatment	1 = resection; 2 = RFA	0.062			
Child–Pugh class	1 = A; 2 = B	0.140			
BCLC stage	1 = 0 or A; 2 = B	0.035	3.412	1.026–11.355	0.045
Total bilirubin, mg/dL		0.571			
Albumin, g/dL		0.301			
Aspartate aminotransferase, IU/L		0.163			
Alanine aminotransferase, IU/L		0.848			
Platelet count, 10^9^/L		0.120			
Prothrombin time, INR		0.296			
MELD score		0.766			
Alpha-fetoprotein, ng/mL		0.329			
PIVKA-II, mAU/mL		0.117			
Baseline IFN-γ producing NK cell proportion, %	1 = < 45%; 2 = ≥ 45%	0.836			
*P1m IFN-γ producing NK cell proportion, %	1 = < 45%; 2 = ≥ 45%	0.001	6.934	2.100–22.897	0.001

^*^P1m, post 1 month after curative treatment; *HCC* hepatocellular carcinoma, *HR* hazard ratio, *CI* confidence interval, *BCLC* Barcelona clinic liver cancer, *IFN* interferon

## Discussion

Defects in NK cell functions have been suggested as important mechanisms for tumor development [37]. However, the association between NK cell dysfunction and HCC progression and recurrence has not been fully evaluated. Especially, little information is currently available regarding post-treatment NK cell function. This prospective study recruited untreated patients with newly diagnosed HCC, and investigated the clinical implication of peripheral blood NK cell activity in HCC stage and recurrence after curative treatment. Our results showed that IFN-γ producing NK cell proportion significantly decreased in patients with intermediate and advanced-stage HCC than very early-stage HCC (42.9% vs. 56.8%, *P* = 0.045). Patients with < 45% IFN-γ producing NK cell proportion at 1 month after treatment exhibited a higher incidence of HCC recurrence than patients with that of ≥ 45% (*P* < 0.001). Moreover, BCLC stage B or C (HR = 3.412) and IFN-γ producing NK cell proportion at 1 month after treatment < 45% (HR = 6.934) independently predicted an increased risk of HCC recurrence (all *P* < 0.05). These findings indicate that NK cell activity decreases in advanced-stage HCC, and patients with decreased NK cell activity at after curative treatment are at higher risk of HCC recurrence even after effective treatment.

Our study has several strengths and clinical implications. First, the association between peripheral blood NK cell activity HCC stage has not been fully clarified in previous studies [19, 20]. In the present study, we observed that, although the total NK cell proportion was rather higher in patients with BCLC BCD than in those with BCLC A HCC (*P* = 0.046), the total IFN-γ producing NK cell proportion was significantly lower in patients with BCLC BCD than in those with BCLC 0 (*P* = 0.045), and a trend of lower IFN-γ producing NK cell proportion was observed in patients with BCLC BCD than in those with BCLC A (*P* = 0.080). These findings indicate that circulating NK cells lose their capacity to produce IFN-γ in advanced HCC, hindering anti-tumor immune response in patients with HCC, and these defects are aggravated with HCC progression regardless of the total NK cell number. It has been well known that NK cells play a major role in initiation and progression of HCC through various mechanisms including decreased frequency and defective cytokine secretion [38]. Cai et al. showed similar result with peripheral blood (IFN-γ producing NK cell proportion of healthy controls vs. Chinese classification Stage I, II, or III, all *P* < 0.05) [20]. However, patients in Chinese classification stage III had higher IFN-γ producing NK cell proportion than those in stage I or II. This disparity may be caused by the difference in HCC staging system. The Chinese classification system was developed in 1999, and not has been widely used for HCC staging.

Second, we found that < 45% IFN-γ producing NK cell proportion at 1 month after curative treatment was independently associated with an increased risk of HCC recurrence (HR = 6.934). Interestingly, the IFN-γ producing NK cell proportion at diagnosis had no significant association with HCC recurrence. This result indicates that restoration of NK cell activity after successful removal of tumor and related microenvironment may play a more critical role in HCC recurrence than decreased NK cell activity at baseline. Previous studies have only evaluated the relationship between NK cell activity at diagnosis and prognosis of patients with HCC. Taketomi et al. showed that ≤ 30% preoperative NK cell activity in PBMCs was independently associated with lower disease-free survival in patients with HCC (*P* = 0.0412) [19]. Although this study suggested important clinical implications, 34.1% of patients had portal vein invasion, and 38.9% of patients had intrahepatic metastasis. It is difficult to believe that hepatectomy could completely remove tumor burden in these patients. Wu et al. reported that the number of NK cells in the intratumoral region is an independent prognostic factor for both overall survival and disease-free survival [21]. The results of our study provide important novel insights not only into the necessity of serial monitoring of immune status in patients with HCC after curative treatment, but also into the rational design and selection of patients for novel immune-based anticancer therapies.

Third, we observed no significant difference in CD56^bright^ NK cell proportion and ratio of CD56^bright^ NK cell and CD56^dim^ NK cell among patients in various stages of HCC (all *P* > 0.05), which is consistent with the findings of Wu et al. and Cai et al. [20, 21]. CD56^dim^ NK cells, which represent mature phenotype of NK cells, mediate cytolytic reaction, in contrast, the immature CD56^bright^ NK cells have been regarded to have cytokine-producing function. Because of the complex interaction between immune cells and cytokines, CD56^bright^ NK cell proportion does not proportionally affect cytokine-producing function. Also, though it has been belived that CD56^dim^ NK cell would decrease in tumor infiltrating lymphocyte compared to non-tumor-infiltrating lymphocyte, there have been conflicting results [20, 21].

We are also aware of several issues that need to be addressed. First, the sample size of this study is small. Further studies with larger number of patients would be needed to validate the results of our study. Second, although we revealed the clinical significance of peripheral blood IFN-γ producing NK cell proportion in HCC, further investigation of liver NK cells is needed to elucidate the immune reaction and specific mechanism involved in NK cell dysfunction. However, although the assessment of NK cell profile in liver tissues provides a more detailed evaluation, histological approach is not easily applicable in clinical setting, especially in serial assessment. Therefore, we assessed NK cell profiles in PBMCs because of the convenience and higher accessibility, and we believe that the results of this study would be helpful in clinical settings. Third, 68.8% of patients had HBV or HCV. It is well known that the status of viral hepatitis affects NK cell activity [39, 40], and this could be a confounding factor. Due to the small sample size, significant correlation between HBV DNA level and NK cell activity may not be found in this study (r = 0.055, *P* = 0.737). Lastly, 19.0% of patients in the curative treatment group were in BCLC stage B, who were not ideal for surgical resection or RFA in most guidelines. However, our findings still revealed that NK cell activity was a more potent predictor for HCC recurrence than HCC stage, and hence, are of great clinical significance.

Consequently, we showed that NK cell activity decreases in advanced-stage HCC. In addition, NK cell activity at 1 month after curative treatment was an independently predictor for increased risk of HCC recurrence. Therefore, serial monitoring of NK cell activity could be helpful in managing patients with HCC. Additionally, our findings could be of clinical importance in the development of novel immune‐based anticancer therapies.

## Supplementary Information

**Additional file 1: Figure 1**. Flowchart of the patient population. HCC, hepatocellular carcinoma; PBMC, peripheral blood mononuclear cell.

**Additional file 2: Figure 2**. FACS plots of patients. FACS, fluorescent‐activated cell sorter; CD, cluster of differentiation.

**Additional file 3: Figure 3**. IFN-γ production of T cell with high IFN-γ (+) (A), low IFN-γ (+) (B), and isotype control (C). FSC-A, forward scatter area; CD, cluster of differentiation; IFN-γ, interferon gamma.

**Additional file 4: Figure 4**. Baseline and 1 month after curative treatment NK cell proportion (A) and IFN-γ producing NK cell proportion (B) of each patient. NK, natural killer, IFN- γ, interferon gamma.

## Data Availability

The datasets used and/or analyzed during the current study are available from the corresponding author on reasonable request.

## Abbreviations

NK: Natural killer
PBMC: Peripheral blood mononuclear cell
HCC: Hepatocellular carcinoma
IFN: Interferon
BCLC: Barcelona clinic liver cancer
HR: Hazard ratio
RFA: Radiofrequency ablation
FACS: Fluorescent‐activated cell sorter
mAb: Monoclonal antibody
CT: Computed tomography
MRI: Multiphase magnetic resonance imaging
HBV: Hepatitis B virus
CI: Confidence interval
