# Supplementary material for: Natural killer cell activity is a risk factor for the recurrence risk after curative treatment of hepatocellular carcinoma
Source: BMC Gastroenterol. 2021 Jun 12;21:258. doi: 10.1186/s12876-021-01833-2 (PMC8199695; doi:10.1186/s12876-021-01833-2)
Supplement: Supplementary file 1 — Additional file 1: Figure 1. Flowchart of the patient population. HCC, hepatocellular carcinoma; PBMC, peripheral blood mononuclear cell. [file 12876_2021_1833_MOESM1_ESM.pdf]

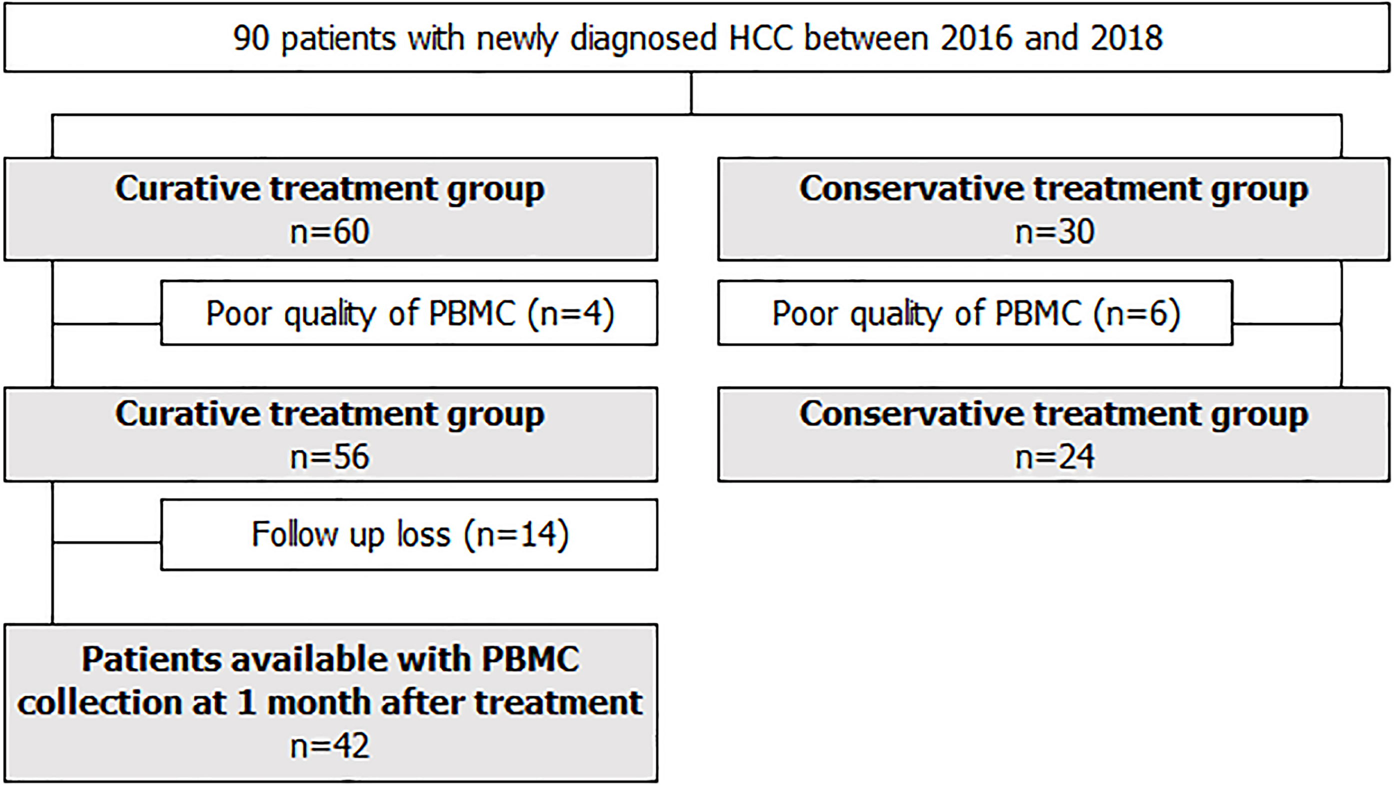
90 patients with newly diagnosed HCC between 2016 and 2018
Curative treatment group
n=60
Conservative treatment group
n=30
Poor quality of PBMC (n=4)
Poor quality of PBMC (n=6)
Curative treatment group
n=56
Conservative treatment group
n=24
Follow up loss (n=14)
Patients available with PBMC collection at 1 month after treatment
n=42
