# Supplementary figures and images for: Natural killer cell activity is a risk factor for the recurrence risk after curative treatment of hepatocellular carcinoma
Source: BMC Gastroenterol. 2021 Jun 12;21:258. doi: 10.1186/s12876-021-01833-2 (PMC8199695; doi:10.1186/s12876-021-01833-2)

CD56

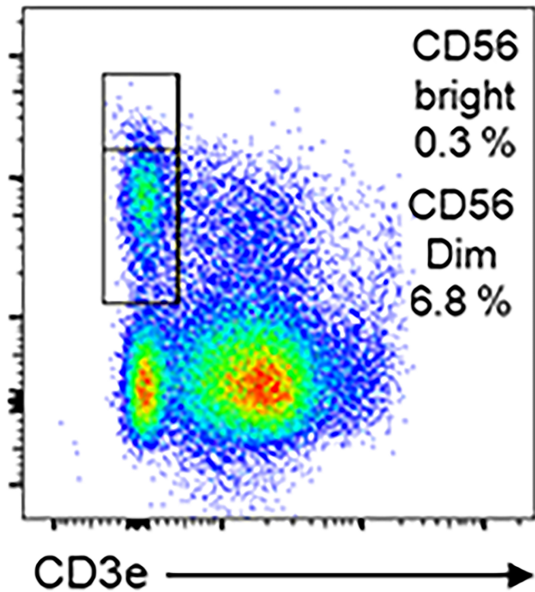

CD56

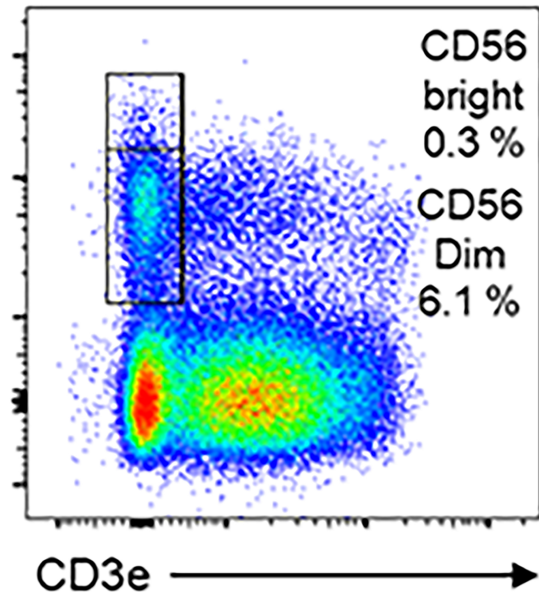

Supplement: Supplementary file 2 — Additional file 2: Figure 2. FACS plots of patients. FACS, fluorescent‐activated cell sorter; CD, cluster of differentiation. [file 12876_2021_1833_MOESM2_ESM.pdf]

(A)

High IFN- $\gamma$

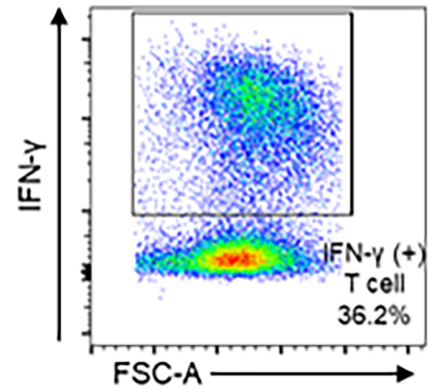

(B)

Low IFN- $\gamma$

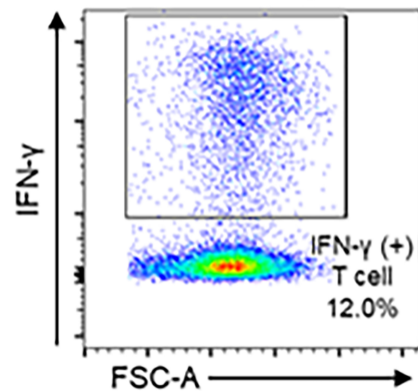

(C)

Isotype

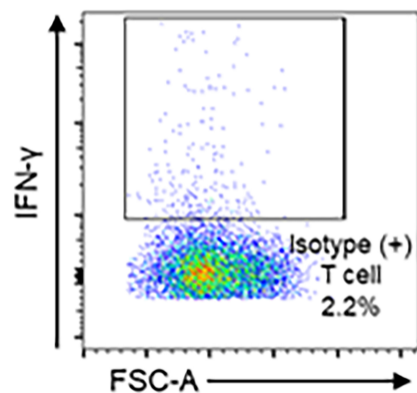

Supplement: Supplementary file 3 — Additional file 3: Figure 3. IFN-γ production of T cell with high IFN-γ (+) (A), low IFN-γ (+) (B), and isotype control (C). FSC-A, forward scatter area; CD, cluster of differentiation; IFN-γ, interferon gamma. [file 12876_2021_1833_MOESM3_ESM.pdf]

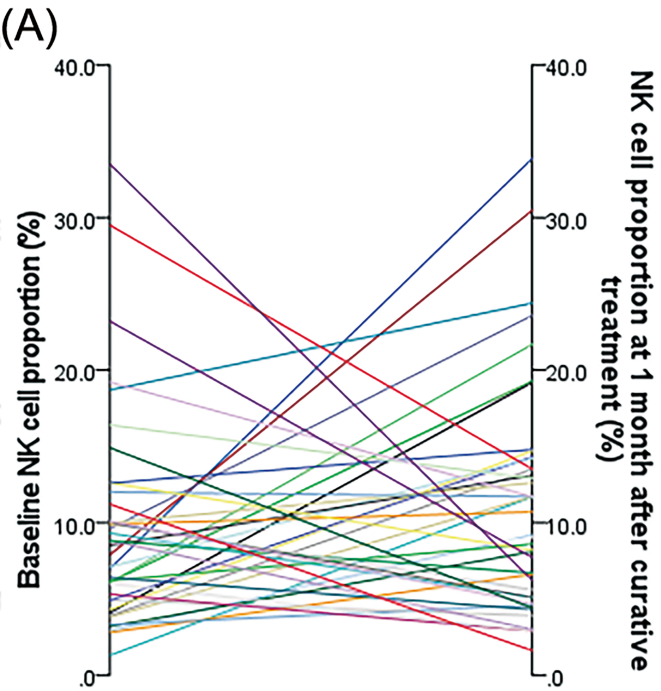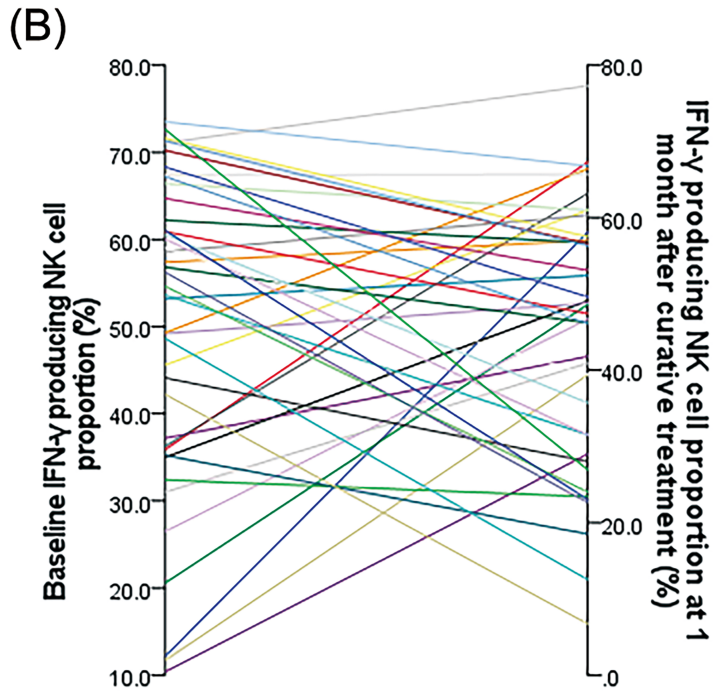

Supplement: Supplementary file 4 — Additional file 4: Figure 4. Baseline and 1 month after curative treatment NK cell proportion (A) and IFN-γ producing NK cell proportion (B) of each patient. NK, natural killer, IFN- γ, interferon gamma. [file 12876_2021_1833_MOESM4_ESM.pdf]
